# 

**Published:** 1882-10-20

**Authors:** 


WM. R. WARNER & CO.—See their new and interesting advertisement in our Journal.

PARKE, DAVIS & CO.—Read the attractive advertisement ot this large establishment in this Journal.

POST-GRADUATE MEDICAL SCHOOL.—See the advertisement of this new Institution in our Journal.

T. GAILLARD THOMAS has accepted the chair of Clinical Professor of Diseases of Women in the College of Physicians and Surgeons, New York.

LACTOPEPTINE.—See new advertisement of New York Pharmacal Association. Their Lactopeptine is now presented in an improved and pure form and free from unpleasant odor and taste. They present it also in a number of combinations, neat and admirably suited to the wants of the practitioner.

THE PEPTONOIDS—as prepared by Reed & Carnrick, we believe to be excellent. We have a number of samples sent us by this excellent house for testing. We think them well worthy of trial.

☞ READ all of our advertisements. They will be found interesting and useful, and do not detract from our reading department which remains uniformly the same in every number.

RECEIPTS will appear in next issue.

